# Study of adiponectin gene (rs1501299) polymorphism and serum adiponectin level in patients with primary knee osteoarthritis

**DOI:** 10.1186/s40246-024-00670-0

**Published:** 2024-09-23

**Authors:** Rehab Elnemr, Mowaffak Moustafa Abd EL Hamid, Raghda Saad Zaghloul Taleb, Naylan Fayez Wahba Khalil, Sherine Mahmoud El-Sherif

**Affiliations:** 1 Department of Physical Medicine, Rheumatology and Rehabilitation, Faculty of Medicine, Alexandria University, Medaan El-Khartoom Square, Al-Azaritah, Alexandria Egypt; 2 Department of Clinical and Chemical Pathology, Faculty of Medicine, Alexandria University, Medaan El-Khartoom Square, Al-Azarita, Alexandria 21561 Egypt

**Keywords:** Adiponectin, Gene polymorphism, Knee, Osteoarthritis, Ultrasonography

## Abstract

**Background:**

We aimed to study, for the first time in the Egyptian population, the relationship between the serum adiponectin level in knee osteoarthritis (KOA) patients and its correlation with clinical, radiological, and ultrasonographic characteristics. Additionally, investigate the relationship between the adiponectin (*ADIPOQ*) gene rs1501299 (+ 276G/T) polymorphism and KOA susceptibility and severity.

**Methods:**

This case-control study enrolled 40 patients with primary KOA and 40 matched controls. All patients underwent physical examination of the knee, pain assessment using the visual analogue scale (VAS), and functional evaluation by Western Ontario and McMaster Universities Osteoarthritis Index (WOMAC). Severity of KOA was assessed by Kellgren Lawrence (KL) grading scale and ultrasonography grading systems. Serum adiponectin levels and adiponectin (*ADIPOQ*) gene single nucleotide polymorphism (SNP) (rs1501299) genotyping were done for all patients and controls.

**Results:**

The study included 40 patients with primary symptomatic KOA and 40 controls with comparable age, sex, and body mass index. The genotype of the rs1501299 (+ 276G/T) polymorphism of the *ADIPOQ* gene was determined using TaqMan allelic discrimination. An enzyme-linked immunosorbent test was used to measure the level of serum adiponectin. The Western Ontario and McMaster Universities Osteoarthritis (WOMAC) score was used to assess functional capability, while the visual analogue scale was utilised to assess knee pain. Using the Kellgren-Lawrence (KL) grading method and global femoral cartilage (GFC) ultrasound grading, the severity of KOA was assessed. No significant differences between patients and controls as regards the genotype distributions and allele frequencies (*p* = 0.400, *p* = 0.507, respectively) of *ADIPOQ* gene rs1501299 (+ 276G/T) polymorphism. Furthermore, serum adiponectin level was significantly higher in the patients compared to healthy subjects (*p* < 0.001). Additionally, adiponectin level had a significant negative correlation with disease severity as evaluated by KL and GFC grading (*r*=-0.351, *p* = 0.027 and *r*=-0.397, *p* = 0.011, respectively).

**Conclusions:**

The *ADIPOQ* gene rs1501299 (+ 276G/T) polymorphism was not associated with KOA severity or vulnerability. The level of adiponectin considerably reduced as the severity of KOA rose, indicating that adiponectin may have a preventive effect in KOA.

## Background

The most prevalent kind of degenerative joint disease, especially in older people, is osteoarthritis (OA) [1]. OA causes pain and functional loss due to irreversible effects such as increasing articular cartilage loss, the formation of osteophytes, and subchondral bone sclerosis [2]. The combination of various factors, including a person’s genetic composition and environmental impacts, causes this complicated condition [3]. Obesity is prevalent in people all over the world, and its expected rise will have an impact on the future incidence of OA, particularly of the knee [4].

Obesity may have a substantial impact on the start and progression of OA in weight-bearing joints by putting an extra mechanical load on joint tissues [5].

The metabolic relationship between OA and obesity may be caused by the pro- or anti-inflammatory characteristics of adipokines [6]. The adiponectin gene (*ADIPOQ*) was found to be responsible for the bulk of circulating adipokines. Adiponectin is produced by the APM1 gene, which has three exons and a length of about 15.8 kb. It is located on chromosome 3q27 and is structurally similar to complement factor C1q, which has been linked to a susceptibility locus for cardiovascular disease, type 2 diabetes, and the metabolic syndrome [7, 8].

Though its role in joint disease is still being debated, it can operate as an anti-inflammatory mediator in a variety of disorders. Other studies have shown that adiponectin promotes inflammation and contributes to the metabolic changes associated with OA. However, because of its capacity to suppress inflammatory responses, it protects against cartilage injury [9]. According to several studies, the level of adiponectin is highly related to the severity of knee OA (KOA) [10]. KOA was most frequently related to the rs1501299 variation in the *ADIPOQ* gene. It could be related to the susceptibility and severity of KOA [11]. This is the first research, as far as we know, into the association between primary KOA and the *ADIPOQ* gene rs1501299 polymorphism in the Egyptian population.

## Subjects and methods

### Study participants

This case-control study was approved by the local Ethics Committee for Human Research, faculty of medicine, Alexandria university (IRB No: 00012098), where a total of 80 participants gave their informed consent before enrollment. They included 40 patients diagnosed with primary KOA based on the American College of Rheumatology (ACR) criteria [12], with 33 females and 7 males. Additionally, 40 healthy individuals were included, who were matched in terms of age, sex, and body mass index (BMI) with the patient group. This control group comprised 31 females and 9 males. All controls lived in the same geographical area with the patients and did not display any symptoms or signs of prior osteoarthritis. The patient recruitment took place at the Rheumatology and Rehabilitation department’s outpatient clinics from August 2020 to April 2021. The minimum sample size was 40 per group determined using the NCSS 2004 and PASS 2000 program to achieve 80% power to detect an effect size of (W = 0.35) [13]. Participants with a history of knee trauma, previous knee surgery, arthritis caused by infection or malignancy, or any other rheumatic diseases were excluded from the study.

All patients underwent complete history taking, anthropometric measurements, thorough physical examination of the knee, pain assessment using the visual analogue scale (VAS), and functional evaluation by Western Ontario and McMaster Universities Osteoarthritis Index (WOMAC) [14]. Plain X-ray was performed for both knees in the standing position and anteroposterior view assessing KOA severity via Kellgren Lawrence (KL) grading scale [15]. Ultrasonography was performed for all patients by a EULAR certified ultrasonographer using a 3–16 MHz linear array transducer (Samsung HS50 ultrasound system, Korea) and the severity of KOA was assessed using ultrasonography grading systems [16].

### Sample collection

Sample collection involved obtaining 10 milliliters of venous blood in the morning after 8 h of fasting, distributed into 4 tubes. Two milliliters were placed in BD Vacutainer^®^ Seditainer™ blacktop blood collection tubes with sodium citrate for measuring erythrocyte sedimentation rate (ESR). Four milliliters were collected in BD Vacutainer^®^ red top blood collection tubes to separate serum for chemical analysis and adiponectin assay. Another 2 milliliters were placed in BD Vacutainer^®^ lavender top blood collection tubes with K3EDTA for a complete blood count (CBC). Lastly, two milliliters were collected in another BD Vacutainer^®^ lavender top blood collection tube with K3EDTA for genotyping the *ADIPOQ* gene single nucleotide polymorphism (SNP) (rs1501299). Serum samples were aliquoted and stored at -80 °C for 3 months. Repeated freeze-thaw cycles were avoided.

### Serum adiponectin measurement

Serum adiponectin levels were measured using the Human Adiponectin ELISA kit (Catalog no.14812, Glory Science Co., Ltd, China). To calculate the adiponectin level in an unknown sample, standard curve was generated.

### Laboratory investigations

Renal function tests (blood urea and serum creatinine) and hepatic function tests (alanine aminotransferase (ALT) and aspartate aminotransferase (AST)) were determined by automated chemistry analyzer Dimension RxL Max (Siemens Healthineers, Germany). CBC was analyzed using ADVIA 2120 (Siemens Healthineers, Germany). C-reactive protein (CRP) was analyzed using the BN Prospec system (Siemens Healthineers, Germany).

### Adiponectin (*ADIPOQ*) gene SNP (rs1501299) genotyping

Genomic DNA was extracted from whole blood via QIAamp DNA Blood Mini Kit (Catalog no. 51104) (QIAGEN, Germany). DNA concentration and purity were measured using a NanoDrop 2000c Spectrophotometer (ThermoFisher Scientific, USA).

Genotyping of *ADIPOQ* gene SNP (rs1501299) was performed by means of TaqMan allelic discrimination assay. The PCR reaction mix contained 10 µL TaqMan^®^ Genotyping Master Mix (2X) (Applied Biosystems-Life Technologies, USA), 1 µL TaqMan^®^ SNP Genotyping Assay 20x (Assay ID: C__7497299_10, Cat. no. 4351379 (Applied Biosystems-Life Technologies, USA), 20 ng DNA/reaction and DNase-free water to reach a final volume of 20 µL. No template control (NTC) containing nuclease-free water was included in each run. Stratagene Mx3000P Q PCR system (Agilent, USA) was programmed as follows: 95 °C for 10 min for AmpliTaq Gold enzyme activation and 40 cycles of denaturation for 15 s at 95 °C and annealing/extension for 1 min at 60 °C. The fluorescence signal was measured at the end of each cycle and a graphic representation of the fluorescence versus the number of cycles was constructed. Data was processed by means of MxPRO qPCR software.

### Statistical analysis

Statistical analyses were conducted using SPSS software version 20.0. (Armonk, NY: IBM Corp). The Kolmogorov-Smirnov was used to test the normality of data distribution. Continuous variables were presented as mean ± standard deviation (SD), whereas categorical variables were presented as number and percentage. The mean of two groups or more were compared using Student’s t-test or one-way analysis of variance (ANOVA) test, respectively, for continuous variables and the χ2-test was used for categorical variables. Differences in allele and genotype frequencies were assessed via Chi-squared (χ2) test. Logistic regression analysis was used to calculate odds ratios (ORs) with 95% confidence intervals (CIs). The Hardy-Weinberg equilibrium was tested using a 𝜒^2^ test. Serum adiponectin level was compared between *ADIPOQ* rs1501299 genotypes and between both study groups using one-way ANOVA and Student’s t-test. Pearson correlation test was used to determine correlations between continuous variables. Multiple linear regression analysis was performed to investigate the association between serum adiponectin and KOA severity, with age, gender, BMI, and disease duration as covariates. A significance level of *p* ≤ 0.05 was considered statistically significant.

## Results

### Characteristics of the study population

Sociodemographic and laboratory criteria of the studied participants are presented in Table 1. The mean age of the studied patients was (49.65 ± 6.84), 21 (52.5%) were housewives, obese patients constituted 67.5% (27 patients). Clinical examination revealed 29 (72.5%) patients had knee effusion, 19 (47.5%) had knee misalignment, 37 (92.5%) had crepitus and 13 (32.5%) had instability. According to KL grading, the mean total KL grading score was 38.83 ± 14.74 ranged from 10.0 to 69.0, the largest percentage of patients had grade II (45%) followed by grade III (40%). Moreover, based on ultrasound findings, the largest percentage of patients (40%) had grade 2b in global femoral cartilage (GFC) grading, followed by grade 2a in 30% of patients.

**Table 1:** Comparison between the two studied groups according to sociodemographic and laboratory characteristics

	Patients (*n* = 40)	Controls (*n* = 40)	Test of significance	*p*
Gender				
Male	7 (17.5%)	9 (22.5%)	χ^2^ = 0.313	0.576
Female	33 (82.5%)	31 (77.5%)		
Age (years)				
Min. – Max.	36.0–70.0	35.0–68.0	t = 1.811	0.074
Mean ± SD.	49.65 ± 6.84	46.90 ± 6.74		
BMI (kg/m^2^)				
Normal weight	1 (2.5%)	2 (5.0%)	χ^2^ = 0.352	^MC^p= 0.839
Overweight	12 (30.0%)	12 (30.0%)		
Obese	27 (67.5%)	26 (65.0%)		
Min. – Max.	24.90–40.60	24.80–43.0	t = 1.833	0.071
Mean ± SD.	32.74 ± 3.88	31.16 ± 3.83		
WHR				
Min. – Max.	0.71– 1.19	0.72–1.28	t = 0.165	0.869
Mean ± SD.	0.87 ± 0.08	0.86 ± 0.11		
ESR (mm/h)				
Min. – Max.	8.0–38.0	3.0–30.0	t = 2.833^*^	0.006^*^
Mean ± SD.	21.83 ± 9.232	16.38 ± 7.928		
CRP (mg/l)				
Min. – Max.	1.2–6.2	0.3–5.3	t = 2.150^*^	0.035^*^
Mean ± SD.	3.3425 ± 1.46075	2.650 ± 1.2195		
Serum adiponectin (ng/ml)				
Min. – Max.	17.47–68.38	3.83–48.07	t = 6.208^*^	< 0.001^*^
Mean ± SD.	46.66 ± 15.70	28.86 ± 9.08		
*ADIPOQ* rs1501299 genotype				
TT	4 (10.0%)	8 (20.0%)	χ^2^ = 1.833	0.400
GT	18 (45.0%)	14 (35.0%)		
GG	18 (45.0%)	18 (45.0%)		
^HW^χ2 (p)	0.026 (0.871)	2.567 (0.109)		
Allele				
T	26 (32.5%)	30 (37.5%)	χ^2^ = 0.440	0.507
G	54 (67.5%)	50 (62.5%)		

SD: Standard deviation, χ^2^: Chi square test, MC: Monte Carlo, t: Student t-test, BMI: body mass index, WHR: waist hip ratio, WOMAC score: Western Ontario and McMaster Universities Osteoarthritis score, KL: Kellgren-Lawrence grading, GFC: Global femoral cartilage grading. p: p value for comparing between the studied groups, ^HW^χ^2^: Chi square for goodness of fit for Hardy-Weinberg equilibrium (If *p* < 0.05 - not consistent with HWE), *: Statistically significant at *p* ≤ 0.05

There was a statistically significant difference between both groups regarding serum adiponectin level (*p* < 0.001), ESR (*p* = 0.006) as well as CRP (*p* = 0.035) being higher in the patient group. On the other hand, there were no statistically significant differences between patients and controls with regard *ADIPOQ* rs1501299 genotype and allele frequencies (*p* = 0.400, 0.507, respectively) (Table 1).

### Association between ADIPOQ rs1501299 polymorphism and risk of KOA

There were no significant associations between *ADIPOQ* rs1501299 polymorphism and all findings of disease characteristics as well as serum adiponectin concentration. Table 2.

**Table 2:** Association between *ADIPOQ* (rs1501299) genotypes and disease characteristics in KOA patients

Variable Mean ± SD	GG (*n* = 18)	GT (*n* = 18)	TT (*n* = 4)	F	*p*
Age (years)	49.77 ± 7.86	48.94 ± 6.38	52.25 ± 3.86	0.375	0.690
BMI (Kg/m^2^)	33.17 ± 3.83	31.80 ± 3.99	34.95 ± 2.98	1.314	0.281
WHR	0.86 ± 0.04	0.87 ± 0.10	0.84 ± 0.10	0.167	0.847
Disease duration (years)	6.22 ± 3.85	6.72 ± 4.89	7.75 ± 2.06	0.223	0.801
VAS	65.00 ± 16.891	72.22 ± 11.144	67.50 ± 5.00	1.246	0.299
WOMAC	36.61 ± 16.103	41.67 ± 14.117	36.00 ± 11.747	0.598	0.555
KL grading	2.28 ± 0.669	2.67 ± 0.840	2.50 ± 0.577	1.224	0.306
GFC grading	2.11 ± 0.758	2.78 ± 1.060	3.00 ± 0.816	3.061	0.059
Serum adiponectin (ng/ml)	50.39 ± 13.58	41.62 ± 16.35	52.48 ± 19.05	1.777	0.183

F: F for ANOVA test p: p value for comparing between different categories, BMI: body mass index, WHR: waist hip ratio, VAS: visual analogue scale, WOMAC score: Western Ontario and McMaster Universities Osteoarthritis score, KL: Kellgren-Lawrence grading, GFC: Global femoral cartilage grading

**Fig. 1:**
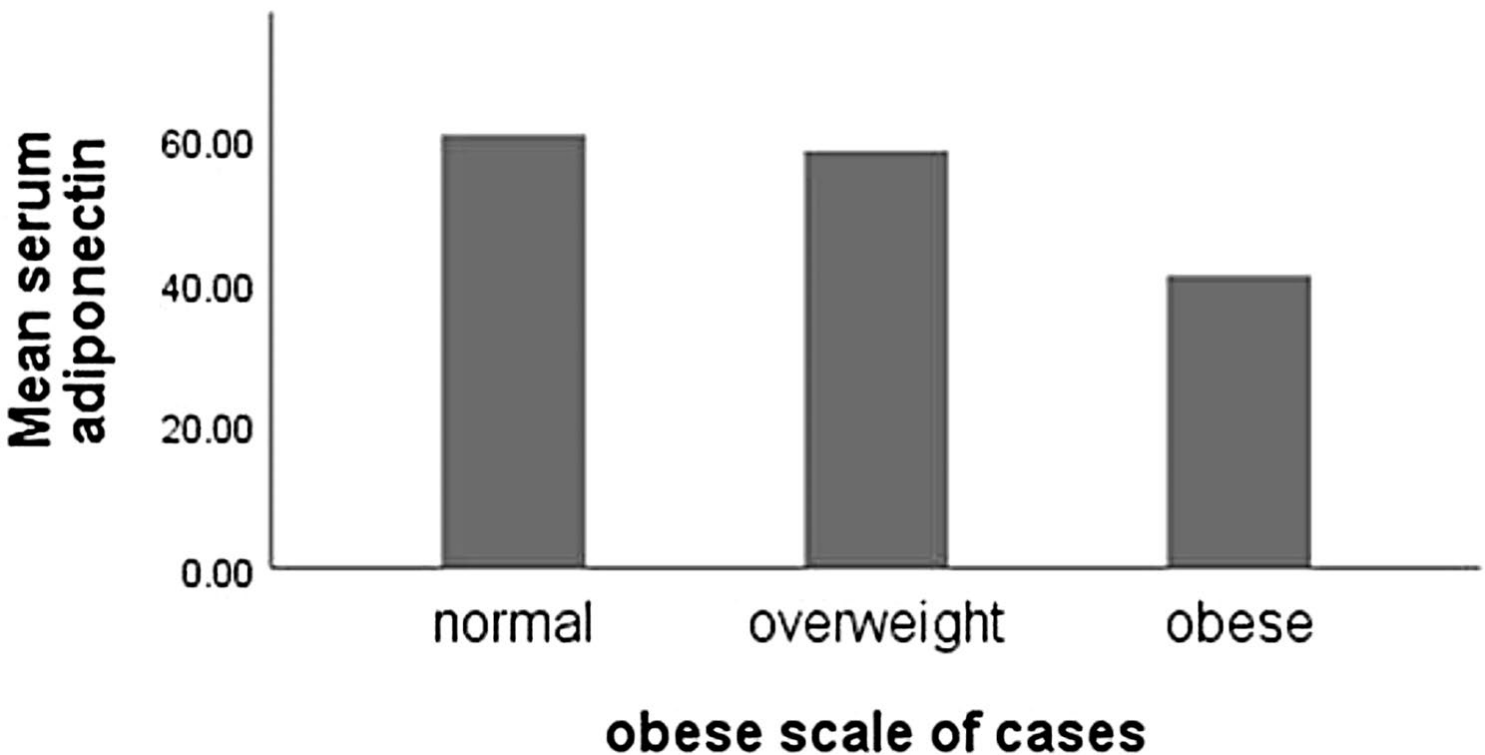
Mean serum adiponectin level according to BMI classification

Correlations between serum adiponectin level and different sociodemographic, clinical, laboratory, radiological, and ultrasonography parameters of studied patients are presented in Table 3. There were significant negative correlations between serum adiponectin level and both BMI (*p* = 0.018) and waist-hip ratio (WHR) (*p* = 0.049). Serum adiponectin concentration had significant negative correlation with neither KL grading (*p* = 0.027) nor GFC grading (*p* = 0.011). The mean serum adiponectin level was significantly lower in obese patients compared to those with overweight and normal weight (*p* = 0.002), Fig. 1.

**Table 3:** Correlation between serum adiponectin level and sociodemographic and disease characteristics

	Serum adiponectin (ng/ml)	Test of Significance	*p*
**BMI (kg/m** ^**2**^ **)**		*r*=-0.373*	0.018*
**Waist circumference**		*r*=-0.229	0.155
**Hip circumference**		*r* = 0.011	0.947
**WHR**		*r*=-0.313*	0.049*
**Effusion**			
Present (*n* = 29)	45.70 ± 16.46	t=-0.620	0.539
Absent (*n* = 11)	49.1736 ± 13.87		
**Wasting**			
Present (*n* = 16)	45.83 ± 15.21	t = 0.017	0.987
Absent (*n* = 24)	46.62 ± 15.28		
**Tenderness**			
Present (*n* = 38)	45.77 ± 15.57	t = 1.579	0.123
Absent (*n* = 2)	63.42 ± 7.00		
**VAS**		*r*=-0.064	0.694
**WOMAC pain subscale**		*r*=-0.161	0.322
**WOMAC stiffness subscale**		*r*=-0.170	0.294
**WOMAC physical function subscale**		*r*=-0.088	0.588
**Total WOMAC score**		*r*=-0.121	0.459
**CRP (mg/l)**		*r* = 0.006	0.970
**ESR (mm/h)**		*r* = 0.012	0.941
**KL grading**		*r*=-0.351^*^	0.027^*^
**GFC grading**		*r*=-0.397^*^	0.011^*^
**MME**		*r*=-0.123	0.450
**LME**		*r*=-0.188	0.245
**Medial osteophytes grading**		*r*=-0.003	0.986
**Lateral osteophytes grading**		*r*=-0.152	0.349

r: pearson correlation, t: Student t-test, p: p value for comparing between the studied groups, *: Statistically significant at *p* ≤ 0.05, BMI: body mass index, WHR: waist hip ratio, VAS: visual analogue scale, WOMAC score: Western Ontario and McMaster Universities Osteoarthritis score, KL: Kellgren-Lawrence grading, GFC: Global femoral cartilage grading, MME: medial meniscal extrusion, LME: lateral meniscal extrusion

Comparisons between serum adiponectin level and different grades of KOA detected by KL or GFC grading on ultrasonography are illustrated in Table 4; Fig. 1. There were significant differences between 4 grades of KOA severity (as detected radiographically or ultrasonographically) regarding serum adiponectin level being lower in higher grades of KOA; the higher the KOA severity, the lower the serum adiponectin level. The regression lines show the trend of serum adiponectin levels to decrease as KL and GFC grades increase, with a decrease of approximately 8.9 and 8.21 units per grade increase in both respectively.

**Table 4:** Comparison between patients with different grades of KOA according to Kellgren-Lawrence grading and global femoral cartilage grading by ultrasound as regards serum adiponectin level

Serum adiponectin (ng/ml)	Kellgren-Lawrence grading of KOA				F	p
	Grade 1 (*n* = 3)	Grade 2 (*n* = 18)	Grade 3 (*n* = 16)	Grade 4 (*n* = 3)		
Min. – Max.	31.20- 68.38	22.14–63.42	20.04–66.19	17.47–27.29	3.622^*^	0.022^*^
Mean ± SD	46.70 ± 19.34	51.52 ± 12.90	45.74 ± 15.82	22.23 ± 4.91		
**Serum adiponectin (ng/ml)**	**Global femoral cartilage grading of KOA**				**F**	**p**
	**Grade 1** **(n = 7)**	**Grade 2a** **(n = 12)**	**Grade 2b** **(n = 15)**	**Grade 3** **(n = 6)**		
Min. – Max.	31.20 -68.38	22.14–65.14	20.04– 66.19	17.47– 43.78	4.636^*^	0.008^*^
Mean ± SD	49.73 ± 14.47	53.53 ± 11.58	46.47 ± 15.86	26.43 ± 10.30		

F: F for ANOVA test, p: p value for comparing between different categories, *: Statistically significant at *p* ≤ 0.05

Comparisons between different *ADIPOQ* rs1501299 genotypes and different grades of KOA as assessed by KL or GFC grading on ultrasonography are illustrated in Table 5.

**Table 5:** Kellgren-Lawrence grading of KOA and global femoral cartilage grading of KOA among the *ADIPOQ* rs1501299 genotypes of studied cases

	GG	GT	TT	χ^2^	(^MC^*p*)
Kellgren-Lawrence grading of KOA					
Grade 1	2	1	0	4.753	0.576
Grade 2	9	7	2		
Grade 3	7	7	2		
Grade 4	0	3	0		
Global femoral cartilage grading of KOA					
Grade 1	4	3	0	8.474	0.205
Grade 2a	8	3	1		
Grade 2b	6	7	2		
Grade 3	0	5	1		

χ ^2^: Chi square test, MC: Monte Carlo test, p: p value for comparison between different categories

After adjusting BMI, age, gender, and duration of the disease on multiple linear regression analysis, there was a significant negative association between serum adiponectin level and KL grading and GFC grading (*p* = 0.041, *p* = 0.005, respectively).

## Discussion

The present study analyzed the association of the *ADIPOQ* gene rs1501299 (+ 276G/T) polymorphism and KOA susceptibility and severity. Such genetic profiling could help identify those who are at a greater risk of developing KOA. According to our results, the allele and genotype frequency of adiponectin + 276G/T polymorphism was not significantly different between KOA patients and controls. Moreover, *ADIPOQ* rs1501299 polymorphism did not affect KOA severity as assessed by KL or GFC ultrasonography grading. Similarly, Zhan et al. [17] in a study on the Thai population revealed no association between the studied polymorphism and increased KOA susceptibility. A Finnish cross-sectional study didn’t find any association between four *ADIPOQ* SNPs and hand OA [18]. On the other hand, a Chinese study was the first to report the relationship between rs1501299 polymorphism and increased risk of OA [11]. Moreover, Zhan et al. [19] from Thailand found a significant association between genotype distribution of + 276G/T polymorphism and KL grade 2, 3 or 4, but with no significant differences in genotype distributions and allele frequencies between KOA patients and controls at two loci of + 45T/G and + 276G/T polymorphisms in the *ADIPOQ*. These contradicting results may be due to the heterogeneous clinical and genetic background between different populations, different sample sizes, genotyping methods, and the interaction between genes and environmental factors.

In the present study, KOA patients had significantly higher serum adiponectin than controls. This suggested that the metabolism of adipose tissue has a significant role in KOA pathogenesis and might probably contribute to its development. Whether a protective or pro-inflammatory contribution, this is still a matter of debate. It has been suggested that adiponectin might be capable of triggering the release of interleukin (IL)-10 and IL-1 receptor antagonists [20]. In addition, it is believed to be implicated in boosting the level of tissue inhibitor of metalloproteinase-2 and suppression of matrix metalloproteinase (MMP)-13 triggered by IL-1β, and consequently antagonize the cartilage destruction pathway [21]. Furthermore, adiponectin was believed to have a role in regulation of inflammation through the reduction of expression of adhesion molecules, and inhibition of macrophage and nuclear factor-kappa B signaling [22]. The assumed protective role of adiponectin in our patients is supported by finding that its serum level was negatively correlated with various indicators of severity suggesting that the higher the level of serum adiponectin, the less severe was the disease. A counterargument may be presented in this context. Adiponectin has been alternatively suggested to promote inflammation and cartilage destruction [23] via destroying chondrocytes and synovial fibroblasts as it can enhance the release of inflammatory mediators including IL-6, IL-8, monocyte chemo-attractant protein-1, MMP-3, and MMP-9 [24]. However, with such destructive potentials, one would expect to observe a positive correlation with the severity of KOA where the opposite has been found in this study.

In support of our results, several studies have shown higher serum levels of adiponectin in patients than controls [9, 25–29]. Some investigators believed that this elevation together with the positive correlation with disease severity supported their assumption of the pro-inflammatory role of adiponectin in OA pathogenesis [28, 30]. On the other hand, others explained this upregulation as a protective compensatory mechanism [9, 26], further supported by its inverse correlation with disease severity.

Interestingly, some data demonstrated that serum and synovial adiponectin levels tended to be lower in OA compared to rheumatoid arthritis [21, 31]. These findings suggested that adiponectin might act as a modulator to slow the progression of arthritis since its expression is higher in “inflammatory arthritis” like rheumatoid arthritis [32].

In the present study, serum adiponectin in KOA patients was inversely correlated with BMI and WHR with no significant correlation with either waist or hip circumferences. This has been also found by others [33] who explained that serum adiponectin is derived mainly from the visceral fat. Previous studies [27, 34, 35] described a similar negative correlation with BMI reinforcing the concept that adiponectin is downregulated in hypertrophic adipose tissue through a negative feedback mechanism [34]. Possible mechanisms could be reduction in insulin-stimulated adiponectin production (as a result of insulin insensitivity), and direct inhibition by TNF-α and IL-6 [36]. Contradictory data have been reported by Koskinen et al. [30] who did not find correlation with neither BMI nor WHR. These discrepancies might be because adiponectin serum level is influenced by other systemic factors, such as nutritional, hormonal and pharmacologic and the possibility of its secretion by tissues other than white adipose tissue [37].

As regards VAS and WOMAC scaling of pain, stiffness and difficulty performing daily activities, we did not find a correlation between serum adiponectin with both pain and all WOMAC subscores and total score. This finding suggested the anti-inflammatory effect of adiponectin and its compatibility with its protective role as proved by others [38]. In contrast, Farag et al. [28] reported that serum adiponectin was significantly associated with pain and all WOMAC scores. These discrepancies might be due to the multiple mechanisms involved with pain which couldn’t be correlated with a single mechanism or a biological marker.

Apart from the KL grading, we assessed KOA severity using GFC grading by ultrasound. Ultrasound can detect minimal structural abnormalities involving articular cartilage, synovial tissue, bony cortex and soft tissues [39]. To the best of our knowledge, this is the first study to use ultrasound to assess severity of KOA in association with serum adiponectin and a negative correlation was found between them. This is consistent with the negative correlation with KL grading. In addition there was no correlation between serum adiponectin level and medial and lateral meniscal protrusion or medial and lateral osteophyte grading or ultrasound detected effusion. Similar findings had been demonstrated by Chen et al. [21], which suggests further that progression of KOA is associated with down-regulation of serum adiponectin. Other studies [25, 28, 40] described contradictory results, with positive correlation of serum adiponectin with disease severity on the basis of its pro-inflammatory role in OA pathogenesis. The explanation for these discrepancies between studies might be due to the small sample size, insufficient control for confounding variables, existence of different detectable adiponectin isoforms with different effects on OA and the use of a low-accuracy radiographic grading method, such as the KL grade.

As the pathogenesis of KOA is not yet fully revealed and no medications could successfully prevent the occurrence or halt the progression of this debilitating disease, the question of adiponectin as a possible pathogenetic or therapeutic factor will still be a matter of debate.

### Study limitations

There were some limitations to this study that should be taken into account. First the small sample size of the study. Second, only one SNP of *ADIPOQ* gene was investigated, which prompted us to go into other SNPs. Third, the effect of *ADIPOQ* gene polymorphism on protein expression was not investigated. Fourth, environmental factors interacting with *ADIPOQ* gene polymorphisms should be investigated in the future. Finally, OA in the hip or hand joints should be researched further.

## Conclusion

According to the results of our study, there was no association between *ADIPOQ* gene rs1501299 (+ 276G/T) polymorphism with KOA susceptibility and severity. Adiponectin level decreased significantly as the severity of KOA increased suggesting that adiponectin may play a protective role in KOA. To fully understand the significance of the adiponectin + 276G/T polymorphism in KOA, and to know more about the potential value of adiponectin as a biochemical determinant of disease progression and prognosis, more research in varied and large populations of KOA patients is needed.

## Data Availability

All data generated or analyzed during this study are included in the published article.

## Abbreviations

ACR: American College of Rheumatology
ALT: Alanine Aminotransferase
AST: Aspartate Aminotransferase
CBC: Complete Blood Count
CRP: C-Reactive Protein
ELISA: Enzyme-Linked Immunosorbent Assay
ESR: Erythrocyte Sedimentation Rate
KL: Kellgren Lawrence
KOA: Knee Osteoarthritis
MMP: Matrix Metalloproteinases
OA: Osteoarthritis
PCR: Polymerase Chain Reaction
VAS: Visual Analogue Scale
WOMAC: Western Ontario and McMaster Universities Arthritis Index

## References

[1] Lee DY. (2024). Prevalence and Risk Factors of Osteoarthritis in Korea: A Cross-Sectional Study. Medicina (Kaunas). Apr 19;60(4):665. 10.3390/medicina60040665 PMC11052057 38674311

[2] Gardiner BS, Woodhouse FG, Besier TF, Grodzinsky AJ, Lloyd DG, Zhang L, Smith DW. Predicting knee osteoarthritis. Ann Biomed Eng. Jan; 2016;44(1):222–33. 26206679 10.1007/s10439-015-1393-5 PMC4690844

[3] Hao Z, Wang Y, Wang L, Feng Q, Li H, Chen T, Chen J, Wang J, Shi G, Chen R, Li B, Zhou S, Jin W, Li J. Burden evaluation and prediction of osteoarthritis and site-specific osteoarthritis coupled with attributable risk factors in China from 1990 to 2030. Clin Rheumatol Jun. 2024;43(6):2061–77. 10.1007/s10067-024-06985-6 38696115

[4] Li H, Kong W, Liang Y, Sun H. Burden of osteoarthritis in China, 1990–2019: findings from the global burden of Disease Study 2019. Clin Rheumatol Mar. 2024;43(3):1189–97. 10.1007/s10067-024-06885-9 PMC10876716 38289570

[5] Yusuf E, Nelissen RG, Ioan-Facsinay A, Stojanovic-Susulic V, DeGroot J, Van Osch G, et al. Association between weight or body mass index and hand osteoarthritis: a systematic review. Ann Rheum Dis. 2010;69:761–5. 19487215 10.1136/ard.2008.106930

[6] Warmink K, Vinod P, Korthagen NM, Weinans H, Rios JL. Macrophage-driven inflammation in metabolic osteoarthritis: implications for Biomarker and Therapy Development. Int J Mol Sci Mar. 2023;24(7):6112. 10.3390/ijms24076112 PMC10094694 37047082

[7] Khabour OF, Alomari MA, Abu Obaid AA. The relationship of adiponectin level and *ADIPOQ* gene variants with BMI among young adult women. Dermatoendocrinol Jun. 2018;18(1):e1477902. 10.1080/19381980.2018.1477902 PMC6298696 30574262

[8] Javor J, Ďurmanová V, Klučková K, Párnická Z, Radošinská D et al. (2024). Adiponectin Gene Polymorphisms: A Case–Control Study on Their Role in Late-Onset Alzheimer’s Disease Risk. *Life 14*, 346. 10.3390/life14030346 PMC10971943 38541671

[9] Tang Q, Hu Z-C, Shen L-Y, Shang P, Xu H-Z, Liu H-X. Association of osteoarthritis and circulating adiponectin levels: a systematic review and meta-analysis. Lipids Health Dis. 2018;17:1–9. 30115130 10.1186/s12944-018-0838-x PMC6097292

[10] Xu H, Kang JH, Choi SE et a. Increased adiponectin levels are associated with higher radiographic scores in the knee joint, but not in the hand joint. Sci Rep. 2021;11:1842. 33469114 10.1038/s41598-021-81513-z PMC7815782

[11] Shang H, Hao Y, Hu W, Hu X, Jin Q. Association between ADIPOQ gene variants and knee osteoarthritis in a Chinese population. Biosci Rep. 2019;39:BSR20182104. 30777928 10.1042/BSR20182104 PMC6395300

[12] Altman R. Criteria for classification of clinical osteoarthritis. J Rheumatol Supplement. 1991;27:10–2. 2027107

[13] Fernández-Torres J, Martínez-Nava GA, Zamudio-Cuevas Y, Martínez-Flores K, Espinosa-Morales R. Epistasis between ADIPOQ rs1501299 and PON1 rs662 polymorphisms is potentially associated with the development of knee osteoarthritis. Mol Biol Rep. 2019;46:2049–58. 30734899 10.1007/s11033-019-04654-5

[14] Guermazi M, Poiraudeau S, Yahia M, Mezganni M, Fermanian J, Elleuch MH, et al. Translation, adaptation and validation of the Western Ontario and McMaster universities osteoarthritis index (WOMAC) for an arab population: the Sfax modified WOMAC. Osteoarthr Cartil. 2004;12:459–68. 10.1016/j.joca.2004.02.006 15135142

[15] JK K. Lawrence JS. Radiological assessment of osteoarthritis. Ann Rheum Dis. 1957;16:494–501. 13498604 10.1136/ard.16.4.494 PMC1006995

[16] Bevers K, Bijlsma JW, Vriezekolk JE, van den Ende CH, den Broeder AA. Ultrasonographic features in symptomatic osteoarthritis of the knee and relation with pain. Rheumatology. 2014;53:1625–9. 24706994 10.1093/rheumatology/keu030

[17] Wang Y, Meng F, Wu J, Long H, Li J, Wu Z, He H, Wang H, Wang N, Xie D. Associations between adipokines gene polymorphisms and knee osteoarthritis: a meta-analysis. BMC Musculoskelet Disord Feb. 2022;22(1):166. 10.1186/s12891-022-05111-4 PMC8864815 35193537

[18] Hämäläinen S, Solovieva S, Vehmas T, Hirvonen A, Leino-Arjas P. Adipokine genes and radiographic hand osteoarthritis in Finnish women: a cross-sectional study. Scand J Rheumatol. 2018;47:71–8. 28812414 10.1080/03009742.2017.1314000

[19] Zhan D, Thumtecho S, Tanavalee A, Yuktanandana P, Anomasiri W, Honsawek S. Association of adiponectin gene polymorphisms with knee osteoarthritis. World J Orthop. 2017;8:719. 28979856 10.5312/wjo.v8.i9.719 PMC5605358

[20] Varra FN, Varras M, Varra VK, Theodosis-Nobelos P. Molecular and pathophysiological relationship between obesity and chronic inflammation in the manifestation of metabolic dysfunctions and their inflammationmediating treatment options (review). Mol Med Rep. Jun; 2024;29(6):95. 38606791 10.3892/mmr.2024.13219 PMC11025031

[21] Luo P, Yuan Q, Wan X, Yang M, Xu P. Effects of Immune cells and cytokines on different cells in OA. J Inflamm Res May. 2023;30:16:2329–43. 10.2147/JIR.S413578 PMC10239267 37273484

[22] Shehzad A, Iqbal W, Shehzad O, Lee YS. Adiponectin: regulation of its production and its role in human diseases. Hormones. 2012;11:8–20. 22450341 10.1007/BF03401534

[23] Ibrahim SM, Hamdy MS, Amer N. Plasma and synovial fluid adipocytokines in patients with rheumatoid arthritis and osteoarthritis. Egypt J Immunol. 2008;15:159–70. 20306680

[24] Bilski J, Schramm-Luc A, Szczepanik M, Mazur-Biały AI, Bonior J, Luc K, Zawojska K, Szklarczyk J. Adipokines in rheumatoid arthritis: emerging biomarkers and therapeutic targets. Biomedicines Nov. 2023;8(11):2998. 10.3390/biomedicines11112998 PMC10669400 38001998

[25] Cuzdan Coskun N, Ay S, Evcik FD, Oztuna D. Adiponectin: is it a biomarker for assessing the disease severity in knee osteoarthritis patients? Int J Rheum Dis. 2017;20:1942–9. 26544540 10.1111/1756-185X.12790

[26] Jiang H, Pu Y, Li ZH, Liu W, Deng Y, Liang R, Zhang XM, Zuo HD. Adiponectin, May be a potential protective factor for obesity-related osteoarthritis. Diabetes Metab Syndr Obes Apr. 2022;27:15:1305–19. 10.2147/DMSO.S359330 PMC9058006 35510046

[27] De Boer T, Van Spil W, Huisman A, Polak A, Bijlsma J, Lafeber F, et al. Serum adipokines in osteoarthritis; comparison with controls and relationship with local parameters of synovial inflammation and cartilage damage. Osteoarthr Cartil. 2012;20:846–53. 10.1016/j.joca.2012.05.002 22595228

[28] Farag ASE-D, Machaly SA, Sultan WA, Soliman NY, Al-Harrass MF, El-Ghaweet AE. Adiponectin in patients with knee osteoarthritis. Benha Med J. 2016;33:133.

[29] Lee YH, Song GG. Association between circulating adiponectin levels and osteoarthritis: a meta-analysis. J Rheumatic Dis. 2018;25:231–8.

[30] Wei G, Lu K, Umar M, Zhu Z, Lu WW, Speakman JR, Chen Y, Tong L, Chen D. Risk of metabolic abnormalities in osteoarthritis: a new perspective to understand its pathological mechanisms. Bone Res Dec. 2023;6(1):63. 10.1038/s41413-023-00301-9 PMC10698167 38052778

[31] Neumann E, Hasseli R, Ohl S, Lange U, Kw F, Muller-ladner U. Adipokines and autoimmunity in inflammatory arthritis. Cells. 2021;10(2):2016. 33499006 10.3390/cells10020216 PMC7910954

[32] Chong T, Tan J, Ann Ma C, Steven, Wong B, Ying-Ying L. Association of adipokines with severity of knee osteoarthritis assessed clinically and on magnetic resonance imaging. Osteoarthr Cartil Open. 2023;5(4):100405. 37664871 10.1016/j.ocarto.2023.100405 PMC10469549

[33] Pilch W, Piotrowska A, Wyrostek J, Czerwińska-Ledwig O, Ziemann E et al. (2022). Different Changes in Adipokines, Lipid Profile, and TNF-Alpha Levels between 10 and 20 Whole Body Cryostimulation Sessions in Individuals with I and II Degrees of Obesity. *Biomedicines*; 10(2):269. 10.3390/biomedicines10020269 PMC8869184 35203477

[34] El Atab O, Ghantous C, El-Zein N, Farahat R, Agouni A, et al. Involvement of caveolae in hyperglycemia-induced changes in adiponectin and leptin expressions in vascular smooth muscle cells. Eur J Pharmacol. 2022;919:174701. 34954233 10.1016/j.ejphar.2021.174701

[35] Askari A, Arasteh P, Homayounfar R, Naghizadeh MM, Ehrampoush E, Mousavi SM, et al. The role of adipose tissue secretion in the creation and pain level in osteoarthritis. Endocr Regul. 2020;54:6–13. 32597150 10.2478/enr-2020-0002

[36] Steyn FCC. (2013) Adiponectin. Handbook of biologically active peptides2 nd ed. San Diego, USA: Elsevier; 2013. pp. 983-9. Academic press.

[37] Wu O, Lu X, Leng J, Zhang X, Liu W, Yang F, Zhang H, Li J, Khederzadeh S, Liu X, Yuan C. Reevaluating Adiponectin’s impact on obesity hypertension: a Chinese case-control study. BMC Cardiovasc Disord. 2024;13(241):208. 10.1186/s12872-024-03865-4 PMC11015577 38615012

[38] Buryanov OA, Kvasha VP, Kupril VO, et al. On the pathogenesis of obesity-associated osteoarthritis. Med Sci Armen. 2023;92(1):60–70.

[39] Podlipská J, Guermazi A, Lehenkari P, Niinimäki J, Roemer FW, Arokoski JP, et al. Comparison of diagnostic performance of semi-quantitative knee ultrasound and knee radiography with MRI: Oulu knee osteoarthritis study. Sci Rep. 2016;6:1–12. 26926836 10.1038/srep22365 PMC4772126

[40] Xu H, Kang JH, Choi SE, Park DJ, Kweon SS, Lee YH, et al. Increased adiponectin levels are associated with higher radiographic scores in the knee joint, but not in the hand joint. Sci Rep. 2021;11:1842. 10.1038/s41598-021-81513-z. 33469114 10.1038/s41598-021-81513-z PMC7815782

